# Antispasmodic Potential of Medicinal Plants: A Comprehensive Review

**DOI:** 10.1155/2021/4889719

**Published:** 2021-11-11

**Authors:** Abdur Rauf, Muhammad Akram, Prabhakar Semwal, Adil A. H. Mujawah, Naveed Muhammad, Zerfishan Riaz, Naveed Munir, Dmitry Piotrovsky, Irina Vdovina, Abdelhakim Bouyahya, Charles Oluwaseun Adetunji, Mohammad Ali Shariati, Zainab M. Almarhoon, Yahia N. Mabkhot, Haroon Khan

**Affiliations:** ^1^Department of Chemistry, University of Swabi, Swabi, Anbar, 23430, Khyber Pakhtunkhwa (KP), Pakistan; ^2^Department of Eastern Medicine, Government College University Faisalabad, Pakistan; ^3^Department of Life Sciences, Graphic Era University, Dehradun 248002, Uttarakhand, India; ^4^Uttarakhand State Council for Science and Technology, Dehradun 248006, Uttarakhand, India; ^5^Department of Chemistry, College of Science and Arts, Qassim University, Ar Rass, 51921, Saudi Arabia; ^6^Department of Pharmacy, Abdul Wali Khan University Mardan, Mardan, 23200, Pakistan; ^7^Department of Biochemistry, Government College University Faisalabad, Pakistan; ^8^K.G. Razumovsky Moscow State University of Technologies and Management (The First Cossack University), 73, Zemlyanoy Val St., Moscow, 109004, Russia; ^9^Laboratory of Human Pathologies Biology, Department of Biology, Faculty of Sciences, and Genomic Center of Human Pathologies, Faculty of Medicine and Pharmacy, Mohammed V University in Rabat, Morocco; ^10^Applied Microbiology, Biotechnology and Nanotechnology Laboratory, Department of Microbiology, Edo University Iyamho, PMB 04, Auchi, Edo State University Uzairue, Nigeria; ^11^Department of Chemistry, College of Science, King Saud University, Riyadh 11451, Saudi Arabia; ^12^Department of Pharmaceutical Chemistry, College of Pharmacy, King Khalid University, Abha, 61421, Saudi Arabia

## Abstract

Numerous medicinal plants have been utilized for the treatment of different types of diseases and disorders including gastrointestinal (GI) diseases. GI diseases are the most common complaints that normally affects the largest proportion of children and adolescents with overlapping clinical manifestation in diagnosis and medical needs. Drugs with antispasmodic effects are normally applied for the symptomatic treatment of contraction and cramping of smooth muscles in gastrointestinal diseases as well as in other critical clinical situations. In alternative system of medicines, the antispasmodic herbs played a significant role in the cure of GI diseases. These medicinal plants and their herbal products are used from generation to generation because of multiple nutritional and therapeutic benefits. The multiple uses might be attributed to the presence on biologically active chemical constitutes. The main aim of this review is to focus on the medicinal potential of plants possessing antispasmodic activities with their proposed mechanism of action. Several databases such as Google Scholar, Cochrane database, Scopus, and PubMed were used to search the relevant literature regarding “plants with antispasmodic activities.” This present study highlights the updated and quantified information on several medicinal plants with antispasmodic activity like *Zanthoxylum armatum*, *Matricaria chamomilla, Foeniculum vulgare*, *Pycnocycla spinosa*, *Atropa belladonna*, *Lavandula angustifolia*, *Mentha pulegium*, *Glycyrrhiza ularensis*, *Anethum graveolens*, and *Origanum majorana*. Moreover, recent studies on other medicinal plant species also have been included in this review article. Additionally, the study also revealed that the active compounds of all these plants possess significant spasmolytic effect which is safest, efficacious, and cost effective as compared to the available synthetic drugs.

## 1. Introduction

Gastrointestinal disorders are common types of complaints that are normally reported among children in most part of the globe. These disorders include functional abdominal pain, ulcerative colitis, irritable bowel syndrome (IBS), infantile colic, and constipation as well as gastroenteritis and acute gastrointestinal disorder. Gastrointestinal disorders can lead to decreased life quality and increased risk of anxiety and depression [1]. These disorders are characterized by recurrent or chronic abdominal pain and in IBS associated with exacerbation or relief by defecation or change in bowel habits [2]. IBS is also associated with abnormalities of intestinal movement together with symptoms such as diarrhea or constipation and pain. People suffering from diarrhea often demonstrate symptoms such as loose, watery stool [3].

IBS occurs all around the world and more common in the United Kingdom, United States, and Scandinavia than other parts of the world with prevalence rate of 40-100 per 100,000 persons and 4-10/100,000 persons per year. IBD is diagnosed most commonly in the mid of 3rd and 4th decades of life without any difference in male and female patients. Family history of IBD was found in about 25% of affected children. However, no differences have been documented with some of these factors such as gender, formula intolerance, breastfeeding, emotional stresses, or prior gastrointestinal illness in between normal and IBD affected children [4]. Ulcerative colitis (UC) is another condition of gastrointestinal abnormalities with confined inflammatory response and morphologic changes to the colon. In UC patients, inflammation is primarily limited to the mucosa, with continuous variation in severity of ulceration, hemorrhage, and edema along with the length of the colon. It has been established during histological examination that acute and chronic inflammation of the mucosa by mononuclear cells, polymorphonuclear leukocytes, goblet cell depletion, distortion of mucosal glands, and crypt abscesses are some of the symptom associated with UP patients [5].

Another inflammatory bowel disease (Crohn's disease) could affect part of the gastrointestinal tract (GIT), starting from the oropharynx to the perianal region. It has transdermal inflammation that could extend to the serosa which often results in sinus tract or fistula formation. Histological study revealed the superficial ulcerations over a Payer's patch and extended chronic inflammation to submucosa [6]. Therefore, this review gave a comprehensive details of gastrointestinal diseases, its pathophysiology, the application of bioactive compounds derived from medicinal plants, and their antispasmodic potential. Additionally, we highlighted a series of 15 traditional medicinal plants having different types of molecules as well as their pharmacological effectiveness.

## 2. Pathophysiology of Gastrointestinal Diseases

There are several mechanisms involved in pathogenesis through which gastrointestinal disorders could be established such as abnormal GI motility and impaired GI mucosa barrier with clinical symptoms of abdominal pain, indigestion, constipation, and diarrhea. The first potential mechanism is the apparently increased risk of overlaps in GI disorders that is linked to reflux. Reflux is the regurgitation of the contents of lower GIT into the upper part of GIT. Disturbed motility has been found in IBS and functional dyspepsia. Reports from several studies have been carried out to validate of the relationship that exist during functional dyspepsia, ulcerative colitis, and IBD with gastro-esophageal reflux disease (GERD) [7, 8]. Moreover, there are several muscles along the GIT which could function as gates or checkpoints which include the orbicular isoris muscle, ileocecal valve, pylorus, proventriculus, and orifice of vermiform appendix, oddi sphincters, and the anus. All these checkpoints are fixed with smooth muscle or sphincter as barrier which resists the opening and effacement challenged due to lower abdominal contents and its failure results in lower gut juice reflux to the upper GIT. Researchers revealed the fluctuation in glucose, GI hormones, and free radicals in oxidative stress in damaged smooth muscle and sphincter patients [9]. The second pathophysiologic mechanism refers to irritable GI stimulation. In GI diseases, the prevalent irritable comorbidity includes depression, anger, and emotional irritation [10]. A nationwide study conducted in Taiwan suggested that using antidepressant agents in the treatment of psychiatric patients portends that capability to increase the risk of upper GI bleeding [7, 11]. Visceral hypersensitivity can be found in irritable GI patients. Moreover, lower sensory threshold for constipation, diarrhea, and abdominal pain has been demonstrated in GI patients most especially those that experience climate change of weather and people that normally consume ice foods [12]. The third mechanism is the stasis of GI microcirculation. Researchers have discovered of fluctuation in catecholamine levels in functional dyspepsia, peptic ulcer, and IBS. Also, some other studies have found disturbed viscosity of blood in colitis and IBS resulting in imbalance of myogenic chemometric autoregulation which leads to the stasis of abdominal circulation [9, 13].

## 3. Traditional Medicines and Antispasmodic Medicinal Plants

Traditional medicines are used since ancient times for curing different diseases. Different parts of the plants are commonly used for the treatment of various illnesses (Tables 1 and 2). Traditional medicines or natural remedies have been identified as a cost-effective method that could be applied for treatment of several diseases [14, 15]. Numerous medicinal herbs have been established to pose a minimal side effect when compared with synthetic drugs [16–18]. These natural therapeutic agents have a lower toxic effects and more economical when compared to synthetic drugs [18, 19]. Typical examples of medicinal plants that could be applied as antispasmodic agent against various GIT disorders like constipation, diarrhea, irritable bowel syndrome (IBS), etc., includes *Ephedra*, *Datura stramonium*, *Solanum dulcamara*, *Atropa belladonna*, *Grindelia camporum*, *Hyssopus officinalis*, *Thymus vulgaris*, *Glycyrrhiza glabra*, *Lobelia inflata*, *Marrubium vulgare*, *Euphorbia hirta*, *Coleus forskohlii*, and *Inula* are the respiratory spasmolytic. Chamomile is an effective anti-inflammatory and antispasmodic traditional medicine used for curing bowel disorders and menstrual discomfort [19, 20]. Spasmolytic compounds are currently used to reduce anxiety, musculoskeletal tension, irritability, and emotional stress. Studies showed that the species belonging to the *Lamiaceae* and *Asteraceae* family have the antispasmodic action with highest number of isolated spasmolytic constituents. Antispasmodic herbs perform their action in different ways [21] such as through inhibition of neurotransmitters like acetylcholine and serotonin or 5-hydroxytryptamine via activation of potassium ATP channels, reduction of extracellular calcium, blocking of muscarine receptors, sodium channels [22, 23], calcium channels, and participation of vanilloid receptors. This is how natural products produce its spasmolytic effect. To check the antispasmodic effects of herbal drugs, variety of experimental models are used which may be the live animals or their organs. When the organ is isolated from the animal, the natural drug or crude extract is administered into it to check the intestinal motility or its action [24]. Medicinal plants are having multi-indications and are used for the treatment and management of various system of the human body [25]. Further, these natural therapeutic remedies are significant sources of various chemical constituents which might be prove new drug candidates [26, 27]. Ample of medicinal plants is used for the treatment of different GIT disorders.

The spasmodic compounds are widely distributed in nature. However, traditional knowledge and ethno-botanical survey provides a base for screening of medicinal plants with antispasmodic potential. Therefore, in this context, we are presenting a quantified and updated information on 15 traditionally high valued medicinal plants having antispasmodic potential through *in vitro* and *in vivo* studies along with clinical trials.

### 3.1. *Matricaria chamomilla* L.

**Figure 1:**
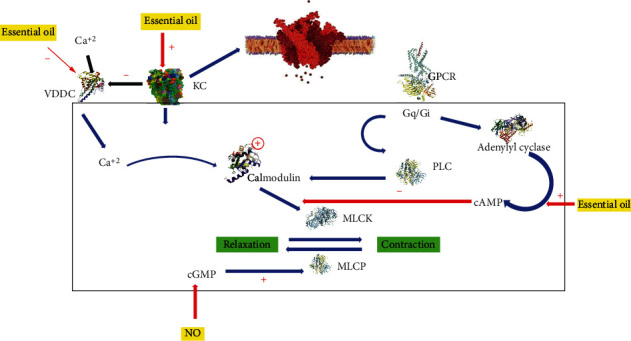
Schematic of molecular antispasmodic mechanism of essential oil. Essential oil inhibited the voltage dependent calcium ion channels and modulated the potassium ion channels and intracellular cyclic adenosine monophosphate. Ca^+2^: calcium ions; VDCC: voltage dependent calcium channel; cGMP: cyclic guanosine monophosphate; GPCR: G protein-coupled receptor; PLC: phospholipase; cAMP: cyclic adenosine monophosphate; MLCK: myosin light chain kinase; MLCP: myosin light chain phosphatase; NO: nitric oxide.

Chamomile is a Greek word which means “ground apple” because it smells like apple. It is the most ancient medicinal herb and is widely used as a tea or tonic. The antispasmodic action of *M. chamomilla* [53, 54] was investigated on isolated ileum of guinea pig and isolated jejunum of rabbit with significant results [55]. The chamomile essential oil is mostly used for its spasmolytic effect. The spasm induced by histamine and acetylcholine was inhibited by the German's chamomile alcoholic extracts. On isolated jejunum of rabbit, myorelaxant effect of alcoholic extract of chamomile was assessed. Due to Ca^+2^ channel blockade [56] and K^+^ channel activation, it causes the relaxation of isolated tissue [57].  Figure 1 depicts the antispasmodic mechanism of essential oil.

### 3.2. *Foeniculum vulgare* Mill

*F. vulgare* commonly called fennel is a perennial herb and flowering plant with feathery leaves and yellow flowers, used in traditional medicine for many ailments. It is one of the oldest medicinal plants in the world. It has many pharmacological properties like anti-inflammatory [58], antibacterial [59, 60], antispasmodic [50], apoptotic, galactagogue, emmenagogue, antioxidant [61], antifungal [62], antimicrobial, and memory enhancing property [63]. It has been established that fennel oil possess a significant antispasmodic or relaxant effect on contractions induced by methacholine tracheal chains of isolated guinea pig. This might be linked to the opening of the potassium channel. The essential oil has beneficial effects on primary dysmenorrhea, and it reduces the pain [64, 65].

### 3.3. *Pycnocycla spinosa* Decne

*P. spinosa* is undomesticated or wild plant which grows in central part of Iran [66]. The essential oil and hydro-alcoholic extracts contain saponin, alkaloids, and flavonoid-rich components having antispasmodic action and antidiarrheal action [67]. The components of *P. spinosa* were split by fraction separation technique to study the relaxant and spasmolytic effect of these three fractions [67, 68]. The hydro-alcoholic extracts of *P. spinosa* were studied on contractions induced in isolated rat ileum. These components of *P. spinosa* showed spasmolytic and relaxant effect and reduced or inhibited the contractions on rat ileum. The main relaxant or spasmolytic effect was shown by alkaloid-rich fraction, and their study showed that *P. spinosa* has the antispasmodic or relaxant effect [69].

### 3.4. *Atropa belladonna* L.

*A. belladonna*, commonly known as deadly nightshade or belladonna, is a perennial herbaceous plant which is poisonous. Its roots and leaves contain alkaloids like scopolamine atropine and hyoscyamine. Because it confuses with other berries, the risk of *A. belladonna* poisoning in children is significant. It is used in both homoeopathy and allopathy, so in allopathy, spasmolytic action is caused by atropine and scopolamine which is the extract of *A. belladonna* and is used in the treatment of colic. It blocks muscarinic receptors which causes smooth muscle relaxation. Scopolamine has potent antispasmodic effect than atropine [70, 71]. The presence of atropine in this plant is a well-known anticholinergic molecule. The antagonistic effect of atropine with muscarinic receptors produce relaxation in GIT muscle which relive spasm and cause inhibition of diarrhea.

### 3.5. *Lavandula angustifolia* Mill

*L. angustifolia* is an evergreen, fragrant, perennial shrub which needs lot of care during fertilizing, sowing, and watering. Its height is about 20 to 100 cm, and its origin is Mediterranean area, i.e., Italy, France, Spain, and Greece. This plant has an antispasmodic activity on rat uterus in vitro and ileum of guinea pig. Previously, the mechanism of action of *L. angustifolia* has not been studied, so the antispasmodic activity was studied on guinea pig ileum *in vitro*. The mechanism of action of *L. angustifolia* was postsynaptic and not atropine-like. The spasmolytic effect of lavender oil was most probably to be transmitted through cAMP and not through cGMP. It has been studied that linalool is one of lavender's major bioactive constituent which has antispasmodic activity [72–74].

### 3.6. *Mentha pulegium* L.

*M. pulegium* is a small plant having dark green or gray leaves. It is locally known as squaw mint or pudding grass or mosquito plant. The plant is known with tubular flowers form summer to autumn. It is also used in Mexican traditional system for the GIT disorders. The essential oil of this plant is highly toxic. The various solvent extracts of *M. pulegium* have been tested for the antispasmodic effect on isolated rat ileum. A significant relaxant effect of the rat intestinal isolated tissues has been published. The dichloromethane fraction was significant spasmolytic due to the antagonistic effect on calcium channels. The study clearly proved that the species of *Mentha* showed antispasmodic effect on smooth muscles [75].

### 3.7. *Glycyrrhiza uralensis* Fisch. ex DC.

This plant is locally known as Chinese liquorice, and the underground part is used as antispasmodic. One of the chemicals constitutes of *G. uralensis* is significant antispasmodic studies reported that glycycoumarin which was isolated from the *G. uralensis* has the antispasmodic activity for carbamylcholine (CCh) induced contractions in jejunum of mouse because it inhibits the phosphodiesterase-III which have inotropic and vasodilative effect. Isoliquiritigenin which is another component of *G. uralensis* was also found as a potent relaxant for KCl, BaCl_2_, and CCh induced contraction, but the amount of isoliquiritigenin was very less in the aqueous extract of *G. uralensis*. So, it was treated with naringinase which increases the amount of isoliquiritigenin and triggered strong antispasmodic activity [76–78].

### 3.8. *Anethum graveolens* L.

*A. graveolens* (family *Apiaceae*) has species that produces dill oil which has fragrant smell and used for lot of disorders [29]. The common ethno-pharmacological uses are antispasmodic and antihiccup in infants. It has muscle relaxant, antiemetic, and anticonvulsant effects. In addition to these folklore, it is also practiced for the treatment of menstrual problems [79, 80]. It reduces flatulence, stomachache, and indigestion [81]. In primary dysmenorrhea, it reduces the pain and has the relaxant effect [82]. Quercetin, rutin, flavonoids, isorhamnetin, and their derivatives are present in dill which is confirmed on phytochemical analysis [83, 84]. The antispasmodic effect on isolated rat ileum is produced by the quercetin and rutin constituents present in dill extract [85].

### 3.9. *Origanum majorana* L.

*O. majorana* is a perennial herbaceous plant and commonly used for the treatment of GIT problems such as stomachache, headache, cold, and fever [25]. Several studies have confirmed its pharmacological properties like antiproliferative [86], antimutagenic [87], antimicrobial [88, 89], antispasmodic [90], antithrombin [91], and antihyperglycemic [92]. Organic fractions of *O. majorana* such as ether, ethyl acetate, petroleum, aqueous extract, methanol, and dichloromethane were investigated for its spasmolytic effect. All these fractions showed significant antispasmodic action especially dichloromethane fraction which has the myorelaxant effect on the isolated jejunum of rabbit.

### 3.10. *Ficus carica* L.

Ficus carica various parts such as fruits, roots, and latex are used in the traditional system. The major chemical metabolites of this medicinal plant are ferulic acid, coumarin, lupeol acetate, sitosterol and other metals, salt and gum, etc. [93]. The ethno-pharmacological uses of *F. carica* include as antitussive, anti-inflammative, appetizer, and antianemic. It is also used locally for the treatment of TB, leprosy, antibacterial, and constipating agent. The plant is also cytotoxic, mild laxative, expectorant, diuretic, and anthelmintic [94]. Antispasmodic activity of *F. carica* has been reported. The aqueous-ethanol extract of this plant was studied. Isolated rabbit jejunum preparations were used for experimental study. Plant extract at dose of 0.1-3.0 mg/mL produced relaxation. Researchers have reported that spasmolytic potential of plant extract is exerted through the activation of K ^(+)^ (ATP) channels [28].

### 3.11. *Satureja hortensis* L.

The major chemical constitutes of *S. hortensis* are carvacrol, terpinene, thymol, and cymene [95]. This medicinal plant is practice locally for the treatment of digestive issues, menstrual problem, respiratory infection, and *Helicobacter pylori* [96]. The biological potentials of this plant include carminative, astringent, aphrodisiac, antispasmodic, and expectorant. Antispasmodic effect of *S. hortensis*'s essential oil in comparison with atropine and dicyclomine has been reported. *S. hortensis* was evaluated for its antispasmodic activity in the contractions of isolated ileum induced by KCl and acetylcholine. The plant extract exhibited dose-dependent response. There was no significant difference in efficacy of *S. hortensis* and dicyclomine. Dicyclomine at dose of 3.46 and 34.6 microgram/mL was able to reduce acetylcholine response on rat-isolated ileum. Atropine also inhibited response to acetylcholine. Their study established that *S. hortensis* extract has relaxant activity that is evident in isolated ileum in rats [42].

### 3.12. *Acorus calamus* L.

*A. calamus* is a very import medicinal plant and used for various disorders, and the major chemical molecules of this plant are alpha-asarone, calamine, methyl isoeugenol, and methyleugenol along with volatile and essential oil [97]. Locally, this plant is used in memory, vascular, and skin problems. This plant is also used for the treatment of digestive- and nervous-related problem [97]. The pharmacological activities of this plant include antispasmodic, anti-inflammatory, antidepressant, antipyretic, antiemetic, expectorant, carminative, tranquillizer, and antibacterial [98]. Spasmolytic potential of *A. calamus* exerted through calcium channel blockage has been reported. Antispasmodic activity of crude extract of *A. calamus* has been conducted using isolated rabbit jejunum preparation. High K^+^ (80 mm) induced contractions were inhibited by use of *A. calamus* and showed antispasmodic activity. Furthermore, tissues were pretreated with plant extract that caused rightward shift in the Ca^+2^ dose-response curves. This effect was similar to standard calcium channel blockers such as verapamil. Further fractionation activity indicated that *n*-hexane fraction is more potent than ethyl acetate fraction. This study indicated that antispasmodic activity of *A. calamus* [99].

### 3.13. *Calendula officinalis* L.

The *C. officinalis* is used for various ailments. Typical examples of the biological constituents present in this plants include calendulin, coumarin, loliolide, and carotenoids [45]. *C. officinalis* is used for various problems such as burn, bleeding of hemorrhoids, fever, cramps, and diabetic foot. It showed hepatoprotective, nephronprotecive, antiedematous, anti-inflammatory, and antioxidant potential [100]. The flowers of *C. officinalis* also possess antispasmodic properties. The aqueous-ethanolic extract of *C. officinalis* was used for antispasmodic activity against isolated gut preparations. The plant extract exhibited dose-dependent (0.03-3.0 mg/mL) relaxation activity in rabbit jejunum. Relaxation activity was mediated via calcium channel blockade [101].

### 3.14. *Carum carvi* L.

The seeds of *C. carvi* possess the following chemical constitutes such as limonene, carvone, and apioe [102]. This plant is used in GIT problems as well as hysteria and convulsion [103]. Its pharmacological activities are carminative, stimulant, aromatic, stomachic, and appetite suppressor [104]. The relaxant potential of alcoholic extract of *C. carvi* on dispersed intestinal smooth muscle cells of the guinea pig has been reported. Ethanolic extract of *C. carvi* was used against dispersed intestinal smooth muscle cells (SMC) of guinea pigs. Micrometric scanning technique was used for assessment of efficacy of *C. carvi* extract on smooth muscle cells and efficacy of acetylcholine on extract pretreated smooth muscle cells. Extracts were used in three different doses (2.5 mg/mL, 250 mg/mL, and 25 mg/mL). Extract was able to reduce the response of dispersed smooth muscle cell to acetylcholine. Smooth muscle cells were pretreated with highest concentration extracts that exhibited less response to acetylcholine. This study indicated the beneficial effects of caraway in reliving gastrointestinal symptoms associated with dyspepsia [105].

### 3.15. *Psidium guajava* L.

The *P. guajava* possess the following chemical constituents such as alpha-pinene, cineol, terpinol, ledol, delinene, ellagic acid, and quercetin [106]. The folklore of this plant is antidiarrheal, anticancer, and antibacterial [33]. The pharmacological activities of *P. guajava* include antispasmodic, antibacterial, and antidysenteric [107]. Antispasmodic potential of the phyto-drug of *P. guajava* folia has been reported. Lozoya and his colleagues performed randomized, double-blind, clinical trials to investigate the potential of a phyto-drug (QG-5) developed from this plant. A total of 50 number of patients (with acute diarrhea) were treated with drug orally (500 mg) up to three days, and capsule was given after every 8 hours. There was significant reduction in frequency of abdominal pain in patients taking *P. guajava* product [108].

There is a limited number of data available on the basis of clinical trials conducted on antispasmodic potential of plants, and it is considered as the weakness of the review. Hence, there is a need for more in-depth researches and clinical trials to understand the potential of these substances, and natural-derived products are also recommended.

## 4. Current Updates: At a Glance

A part from this, recently, few authors also demonstrated the antispasmodic potential of other plants and regarding this from Algeria, reported about the inflammatory activity, antispasmodic activity, and healing activity of *Juglans regia* L. using *in vivo* models [109]. The Carrageenan-induced paw edema test, acetic acid-induced endogenous spasm test, and the healing effects were performed. The results indicated that plant extract possesses strong anti-inflammatory (extract: 25% and diclofenac: 31%), antianalgesic (extract: 60% and Spasfon: 83%), and healing (extract: 7 days and madecassol: 9 days) properties when compared with the control [109]. The detailed investigation on antispasmodic, antidepressant, and anxiolytic effects of medicinal plant (*Schinus lentiscifolius*) from Argentina has been documented [110]. The plant bioactive compound (*S. lentiscifolius* tincture) displayed concentration-dependent antispasmodic activity in intestinal muscle (IC_50_: 6.32 mg SchT/mL), bladder (IC_50_: 2.63 mg SchT/mL), and uterus (IC_50_: 4.05 mg SchT/mL) with a noncompetitive antagonism on the cholinergic contraction [110].

Digas colic drops (DCD-684), a poly herbal formulation of five different medicinal plants (*Carum carvi* L., *Foeniculum vulgare* Mill., *Mentha arvensis* L., *Mentha piperita* L., and *Zingiber officinale* Roscoe), were evaluated for their spasmolytic effects (spontaneous and precontracted tissues). DCD-684 showed spasmolytic effects on both spontaneous (IC_50_: 0.75%) and KCl-induced contractions (IC_50_: 1.6%), respectively. The DCD-684 demonstrated synergistic effects due to the presence of different bioactive compounds and its proposed molecular mechanism of action governed by muscarinic and/or nicotinic receptors, serotonergic histaminergic, along with calcium channel blocking mechanisms [111]. While in another study, Parcha et al. reported about the papaverine (isolated from *Papaver somniferum*) and its synergistic effects with known drug imatinib against chronic myeloid leukemia [112].

The spasmolytic activity of polyherbal formulations of three plant species (*Apium graveolens* L., *Helicteres isora* L., *Mentha piperita* L.) was evaluated on guinea pig ileum model. The results indicated that polyherbal formulations displayed significant reduction of spasm induced by acetyl choline, nicotine, and histamine in guinea pig ileum. Additionally, intestinal motility in mice also inhibited by the polyherbal formulation and proposed molecular mechanism governed by the inhibition of muscarinic, nicotinic, and histamine receptors [113]. The plant extract of *Nephelium lappaceum* L. was evaluated for spasmolytic activity against isolated chicken ileum. The acetylcholine and histamine were used to trigger the contractile response, and atropine, mepyramine, and plant extract were used to reduce the intestinal contractions. The plant extract showed antimotility and antispasmodic properties by blocking the acetylcholine and histamine [114]. Additionally, few other authors also describe the antispasmodic potential of plant species from different regions [115–125].

The antispasmodic therapeutic agents are the remedies that could lead to higher level of relive the spam of GIT muscle and also attenuating the liquid component of GIT to block diarrhea. The spasm is induced by the stimulation of cholinergic/muscarinic, opioids, and histaminic receptors. These receptors are present in the GIT as well as in other body parts. The stimulation of cholinergic receptors (M_1_, M_3_, and M_5_) leading to stimulate the intracellular signaling up to activation of PKA and PKC. Both of these kinase enzymes stimulate the calcium channel and increasing intracellular calcium by increasing the calcium influx. The intracellular calcium is also increasing by the opening of calcium gates at ER and release calcium to the cytoplasm. This increased calcium level interacts with smooth muscle (SM) and causes full contraction which also produce diarrhea. This is the basic mechanism in the antispasmodic experiments. In the above discussion of medicinal plants showing antispasmodic action, the plant constituents or the extract/fraction blocked these receptors. Once these receptors are blocked, the cAMP level will significantly drop, and PKA/PKC will inhibited resulting decrease intracellular calcium. This decrease calcium level is now not sufficient to contract the SM but will lead to relaxation of SM. This relaxant effect on SM is known as antispasmodic effect or antidiarrheal effect. In this experiment, calcium is used for the induction of spasm in SM keeping in mind the above mechanism. Some researcher uses histamine for contraction of SM because histamine stimulate the H_1_ or H_2_ receptors. Both of these receptors are stimulatory and increasing the cAMP level which keep on PKA stimulated. This stimulated kinase increases calcium influx and causes contraction. Various plants inhibited this histamine induced contraction due to antagonistic effect on H_1_ or H_2_ receptors. In case of opioid receptor stimulation, the intracellular signaling is different from the above mechanism because opioid receptors are inhibitory receptors, so the stimulation of these inhibitory receptors will decrease the cAMP level, but the dimer of beta and gamma will stimulate the potassium channel leading to potassium efflux. This cation efflux will cause depolarization, and the SM will relax. Morphine is one of the major constituent of opium plant which causes agonistic effect on opioid receptors. In the antispasmodic experiments, the potassium is used to check the inhibitory phenomenon.

## 5. Conclusion

It might be concluded on the basis of obtained information that the spasmodic pain can be treated by traditional natural medicines which are effective, safe, and economical. Under this review, many plant species have been identified that reduces the spasmodic pain with low toxicity and side effects. The recent research data supports the use of herbal medicines for treatment of variety of ailments. Hence, more work is being carried out to identify the potential antispasmodic effect of different plant species which deserves more awareness in terms of *in vivo* and clinical trial studies. The detailed literature analysis of medicinal plants could be the rich source of antispasmodic compounds and opened up new areas for researchers and scholars in this field. Therefore, further pharmacokinetics and pharmacodynamics along with clinical trials should be carried out to validate the efficacy and reveal the safety profile of traditional medicines to manage spasmodic disorders.

## Figures and Tables

**Table 1:** An overview of antispasmodic plants, used parts, chemical constituents, and tested models.

	Antispasmodic plants	Compound name	Part used with solvent	Tested models	References
1	*Glycyrrhiza uralensis*	Glycycoumarin	Root infusion (aqueous)	Caracole in mouse jejunum	[28]
2	*Plectranthus barbatus*	Myrcene	Leaf (MeOH)	KCl, ACh, BaCl_2_, in guinea pig ileum	[29]
3	*Nepeta cataria*	Geranyl acetate	Leaf (aqueous)	KCl in guinea pig trachea and rat jejunum	[30]
4	*Cynara scolymus*	Cynaropicrin	Leaf, flower infusion (MeOH)	ACh in guinea pig	[31]
5	*Artemisia vulgaris*	Ezozlantonin	Leaf (CHCl_3_)	PMA, S, and H in guinea pig trachea and ileum	[32]
6	*Thymus vulgaris*	Thymol	Whole plant (ethanol)	BaCl_2_, KCl, and ACh in rat trachea and ileum	[33]
7	*Radix aucklandiae*	Costunolide	Rhizome (MeOH)	KCl, S, and ACh in rat jejunum	[34]
8	*Allium elburzense*	Agapanthagenin	Bulb and flower infusion (hexane)	H in guinea pig ileum	[35]
9	*Eucalyptus camaldulensis*	*β*-Sitosterol	Leaf infusion (EtOAc)	Spontaneous contraction in rabbit jejunum (KCl)	[36]
10	*Allium cepa*	Tropeoside B1 and B2	Bulbs, 9 : 1 (CHCl_3_:MeOH)	ACh and H in guinea pig ileum	[37]
11	*Zygophyllum gaetulum*	Zygophyloside N	Root infusion (MeOH)	Isolated guinea pig ileum (electrically induced contractions)	[38]
12	*Galphimia glauca*	Galphimin F	Leaf infusion (MeOH)	In guinea pig ileum (electrical-induced contraction)	[39]
13	*Tylophora hirsuta*	*α*-Amyrin acetate	Aerial parts (MeOH)	KCl in rabbit jejunum	[40]
14	*Prangos ferulacea*	Osthole	Root (acetone)	KCl, ACh, and electric field stimulation in rat ileum	[41]
15	*Hypericum perforatum*	Hypericin	Aerial parts (EtOH 70%)	KCl in rabbit jejunum	[42]
16	*Pycnocycla spinosa*	Isovanillin	Aerial parts (MeOH)	KCl in rat ileum	[43]
17	*Anethum graveolens*	Dill	Dill fruit hydro-alcoholic extract	Methacholine tracheal chains of isolated guinea pig	
18	*Matricaria recutita*	Chamazulene	Plant infusion (aqueous)	Human platelet	[44]
19	*Viburnum prunifolium*	Patrinoside	Root and stem bark infusion (MeOH)	Caracole in guinea pig trachea and in rat jejunum	[45]
21	*Valeriana procera*	Valtriate	Root infusion (EtOH)	KCl, BaCl_2_ carbachol in guinea pig ileum and stomach	[46]
22	*Zingiber officinale*	Phellandrene	Rhizome infusion	S in rat ileum	[47]
23	*Artemisia monosperma*	Sternbin	Aerial part (EtOH)	ACh and O in rat ileum, urinary bladder, trachea, pulmonary artery, and uterus	[48]
24	*Drosera rotundifolia*	Quercetin	Dried aerial parts (aqueous and ethanolic extract)	Guinea pig ileum	[49]
25	*Tamarindus indica*		Methanolic extract of fruits	KCl-induced contraction of rabbit jejunum	[50]
26	*Moringa oleifera*	Niazinin	Seed infusion, ethanolic extract of *Moringa oleifera* leaves	The acetylcholine-induced contraction isolated guinea pig atria	[51]
27	*Matri cariarecutita* L.	Flavonoids	Chamomile's alcoholic extract	On isolated jejunum of rabbit	[52]

MeOH: methanol; CHCl_3_: chloroform; EtOAc: ethyl acetate; ACh: acetylcholine; BaCl_2_: barium chloride; KCl: potassium chloride; O: oxytocin; PMA: *β*-phenylethyl amsine; PGF: prostaglandin F2*α*; H: histamine; S: serotonin.

**Table 2:** Antispasmodic mechanism of actions of phytomedicines.

Phytomedicines	Mechanism of action	References
*Glycyrrhiza uralensis*	Inhibitory action of acetylcholine and histamine-induced contractions	[28]
*Plectranthus barbatus*	Acetylcholine inhibition	[29]
*Nepeta cataria*	Cholinergic effect, calcium channel blockade	[30]
*Cynara scolymus*	Acetylcholine inhibition	[31]
*Artemisia vulgaris*	Blockade of muscarinic receptors and calcium influx	[32]
*Thymus vulgaris*	Effect on anticholinergics and serotonergic pathways	[33]
*Radix aucklandiae*	Inhibition of muscarinic receptors and calcium influx	[34]
*Allium elburzense*	Decrease the activities of methanogenesis	[35]
*Eucalyptus camaldulensis*	Inhibit the formation of prostaglandins and cytokines	[36]
*Allium cepa*	Spasmolytic effect via calcium channels	[37]
*Zygophyllum gaetulum*	Inhibition of muscarinic receptor and calcium influx	[38]
*Galphimia glauca*	GABAergic effect	[39]
*Tylophora hirsuta*	Calcium channel blockade	[40]
*Prangos ferulacea*	Inhibit the response of KCl by calcium channel blockage	[41]
*Hypericum perforatum*	Reduces intestine motility	[42]
*Pycnocycla spinosa*	Inhibitory effect on both KCl and EFS responses	[43]
*Anethum graveolens*	Potassium channel opening	
*Matricaria recutita*	Ca^+2^ channel blockage, NO release, ACh receptors, and PKA_2_ activation	[44]
*Viburnum prunifolium*	Effects on cholinesterase	[45]
*Valeriana procera*	Block the autonomic receptors, acting as musculotropic agents	[46]
*Zingiber officinale*	Antihistaminergic, antiserotonergic	[47]
*Artemisia monosperma*	Changes in Ca^+2^ metabolism	[48]
*Drosera rotundifolia*	Affecting allosteric binding site of the muscarinic M3 receptors, inhibit neutrophil elastase	[49]
*Tamarindus indica*	Calcium channel blockade	[50]
*Moringa oleifera*	Inhibited acetylcholine-induced contractions	[51]
*Matricaria recutita*	Calcium channel blockade	[52]

## Data Availability

The available data are presented in the text of this manuscript.

## References

[1] Anheyer D., Frawley J., Koch A. K. (2017). Herbal medicines for gastrointestinal disorders in children and adolescents: a systematic review. *Pediatrics*.

[2] Holtmann G., Shah A., Morrison M. (2017). Pathophysiology of functional gastrointestinal disorders: a holistic overview. *Digestive Diseases*.

[3] Sadraei H., Ghanadian M., Asghari G., Madadi E., Azali N. (2014). Antispasmodic and antidiarrhoeal activities of 6-(4-hydroxy-3-methoxyphenyl)-hexanonic acid from Pycnocycla spinosa Decne. exBoiss. *Research in Pharmaceutical Sciences*.

[4] Ananthakrishnan A. N. (2015). Epidemiology and risk factors for IBD. *Nature Reviews Gastroenterology & Hepatology*.

[5] Colitis–Pathophysiology, U (2003). Inflammatory bowel disease part I: ulcerative colitis–pathophysiology and conventional and alternative treatment options. *Alternative Medicine Review*.

[6] Hendrickson B. A., Gokhale R., Cho J. H. (2002). Clinical aspects and pathophysiology of inflammatory bowel disease. *Clinical Microbiology Reviews*.

[7] Lee Y.-C., Shau W. Y., Chang C. H., Chen S. T., Lin M. S., Lai M. S. (2012). Antidepressant use and the risk of upper gastrointestinal bleeding in psychiatric patients: a nationwide cohort study in Taiwan. *Journal of Clinical Psychopharmacology*.

[8] Yarandi S. S., Christie J. (2013). Functional dyspepsia in review: pathophysiology and challenges in the diagnosis and management due to coexisting gastroesophageal reflux disease and irritable bowel syndrome. *Gastroenterology Research and Practice*.

[9] Ling W., Li Y., Jiang W., Sui Y., Zhao H. L. (2015). Common mechanism of pathogenesis in gastrointestinal diseases implied by consistent efficacy of single Chinese medicine formula: a PRISMA-compliant systematic review and meta-analysis. *Medicine*.

[10] van Oudenhove L., Vandenberghe J., Demyttenaere K., Tack J. (2010). Psychosocial factors, psychiatric illness and functional gastrointestinal disorders: a historical perspective. *Digestion*.

[11] Lee S. Y., Lee K. J., Kim S. J., Cho S. W. (2009). Prevalence and risk factors for overlaps between gastroesophageal reflux disease, dyspepsia, and irritable bowel syndrome: a population-based study. *Digestion*.

[12] Bredenoord A. J., Chial H. J., Camilleri M., Mullan B. P., Murray J. A. (2003). Gastric accommodation and emptying in evaluation of patients with upper gastrointestinal symptoms. *Clinical Gastroenterology and Hepatology : the Official Clinical Practice Journal of the American Gastroenterological Association*.

[13] Mitsuyama K. (2009). Therapeutic leukocytapheresis in inflammatory bowel disease: clinical efficacy and mechanisms of action. *Cytotherapy*.

[14] Ghasemi P. A., Momeni M., Bahmani M. (2013). Ethnobotanical study of medicinal plants used by Kurd tribe in Dehloran and Abdanan districts, Ilam Province, Iran. *African Journal of Traditional, Complementary, and Alternative medicines : AJTCAM*.

[15] Tang S. Y., Halliwell B. (2010). Medicinal plants and antioxidants: What do we learn from cell culture and *Caenorhabditis elegans* studies?. *Biochemical and Biophysical Research Communications*.

[16] Beyrami-Miavagi A., Farokhi F., Asadi-Samani M. (2014). A study of the effect of prostodin and hydroalcoholic extract of Malva neglecta on kidney histopathology and renal factors in female rats. *Advances in Environmental Biology*.

[17] Kooti W., Ghasemiboroon M., Asadi-Samani M. (2014). The effect of halcoholic extract of celery leaves on the delivery rate (fertilization and stillbirths), the number, weight and sex ratio of rat off spring. *Advances in Environmental Biology*.

[18] Kooti W., Mansouri E., Ghasemiboroon M., Harizi M., Ashtary-Larky D., Afrisham R. (2014). The effects of hydroalcoholic extract of Apium graveolens leaf on the number of sexual cells and testicular structure in rat. *Jundishapur Journal of Natural Pharmaceutical Products*.

[19] Asadi-Sama M., Rafieian-K M., Azimi N. (2013). Gundelia: a systematic review of medicinal and molecular perspective. *Pakistan Journal of Biological Sciences*.

[20] Nathan M., Scholten R. (1999). *The Complete German Commission e Monographs: Therapeutic Guide to Herbal Medicines*.

[21] Taqvi S. I. H., Shah A. J., Gilani A. H. (2009). Insight into the possible mechanism of antidiarrheal and antispasmodic activities of piperine. *Pharmaceutical Biology*.

[22] Gálvez J., Sánchez de Medina F., Jiménez J., Zarzuelo A. (2001). Effects of flavonoids on gastrointestinal disorders. *Studies in Natural Products Chemistry*.

[23] Mehmood M. H., Siddiqi H. S., Gilani A. H. (2011). The antidiarrheal and spasmolytic activities of Phyllanthus emblica are mediated through dual blockade of muscarinic receptors and Ca^2+^ channels. *Journal of Ethnopharmacology*.

[24] Martínez-Pérez E. F., Juárez Z. N., Hernández L. R., Bach H. (2018). Natural antispasmodics: source, stereochemical configuration, and biological activity. *BioMed Research International*.

[25] Farnsworth N., Wilson E., Peter F. M. (1988). Screening Plants for New Medicines. *Biodiversity*.

[26] Blumenthal M., Goldberg A., Brinckmann J. (2000). *Herbal medicine. Expanded commission E monographs*.

[27] Pimple B., Patel A. N., Kadam P. V., Patil M. J. (2012). Microscopic evaluation and physicochemical analysis of *Origanum majorana* Linn leaves. *Asian Pacific Journal of Tropical Disease*.

[28] Nagai H., Yamamoto Y., Sato Y., Akao T., Tani T. (2006). Pharmaceutical evaluation of cultivated Glycyrrhiza uralensis roots in comparison of their antispasmodic activity and glycycoumarin contents with those of licorice. *Biological and Pharmaceutical Bulletin*.

[29] Callan N. W., Johnson D. L., Westcott M. P., Welty L. E. (2007). Herb and oil composition of dill (*Anethum graveolens* L.): Effects of crop maturity and plant density. *Industrial Crops and Products*.

[30] Gilani A. H., Shah A. J., Zubair A. (2009). Chemical composition and mechanisms underlying the spasmolytic and bronchodilatory properties of the essential oil of *Nepeta cataria* L.. *Journal of Ethnopharmacology*.

[31] Emendörfer F., Emendörfer F., Bellato F. (2005). Antispasmodic activity of fractions and cynaropicrin from Cynara scolymus on guinea-pig ileum. *Biological and Pharmaceutical Bulletin*.

[32] Natividad G. M., Broadley K. J., Kariuki B., Kidd E. J., Ford W. R., Simons C. (2011). Actions of *Artemisia vulgaris* extracts and isolated sesquiterpene lactones against receptors mediating contraction of guinea pig ileum and trachea. *Journal of Ethnopharmacology*.

[33] Begrow F., Engelbertz J., Feistel B., Lehnfeld R., Bauer K., Verspohl E. (2010). Impact of thymol in thyme extracts on their antispasmodic action and ciliary clearance. *Planta Medica*.

[34] Guo H., Zhang J., Gao W., Qu Z., Liu C. (2014). Gastrointestinal effect of methanol extract of Radix Aucklandiae and selected active substances on the transit activity of rat isolated intestinal strips. *Pharmaceutical Biology*.

[35] Barile E., Capasso R., Izzo A. A., Lanzotti V., Sajjadi S. E., Zolfaghari B. (2005). Structure-activity relationships for saponins from Allium hirtifolium and Allium elburzense and their antispasmodic activity. *Planta Medica*.

[36] Begum S., Sultana I., Siddiqui B. S., Shaheen F., Gilani A. H. (2002). Structure and spasmolytic activity of eucalyptanoic acid from Eucalyptus camaldulensis var. obtusa and synthesis of its active derivative from oleanolic acid. *Journal of Natural Products*.

[37] Corea G., Fattorusso E., Lanzotti V., Capasso R., Izzo A. A. (2005). Antispasmodic saponins from bulbs of red onion, Allium cepa L. var. Tropea. *Journal of agricultural and food chemistry*.

[38] Aquino R., Tortora S., Fkih-Tetouani S., Capasso A. (2001). Saponins from the roots of *Zygophyllum gaetulum* and their effects on electrically-stimulated guinea-pig ileum. *Phytochemistry*.

[39] González-Cortazar M., Tortoriello J., Alvarez L. (2005). Norsecofriedelanes as spasmolytics, advances of structure-activity relationships. *Planta Medica*.

[40] Pavlović I., Krunić A., Nikolić D. (2014). Chloroform extract of underground parts of Ferula heuffelii: secondary metabolites and spasmolytic activity. *Chemistry & Biodiversity*.

[41] Sadraei H., Shokoohinia Y., Sajjadi S., Mozafari M. (2013). Antispasmodic effects of Prangos ferulacea acetone extract and its main component osthole on ileum contraction. *Research in Pharmaceutical Sciences*.

[42] Khan A., Gilani A. H., Najeeb-ur-Rehman (2011). Pharmacological studies on Hypericum perforatum fractions and constituents. *Pharmaceutical Biology*.

[43] Sadraei H., Ghanadian M., Asghari G., Azali N. (2014). Antidiarrheal activities of isovanillin, iso-acetovanillon and Pycnocycla spinosa Decne ex. Boiss extract in mice. *Research in pharmaceutical sciences*.

[44] Maschi O., Cero E. D., Galli G. V., Caruso D., Bosisio E., Dell’Agli M. (2008). Inhibition of human cAMP-phosphodiesterase as a mechanism of the spasmolytic effect of Matricaria recutita L. *Journal of Agricultural and Food Chemistry*.

[45] Cometa M. F., Parisi L., Palmery M., Meneguz A., Tomassini L. (2009). *In vitro* relaxant and spasmolytic effects of constituents from *Viburnum prunifolium* and HPLC quantification of the bioactive isolated iridoids. *Journal of Ethnopharmacology*.

[46] Hazelhoff B., Malingré T. M., Meijer D. K. (1982). Antispasmodic effects of valeriana compounds: an in-vivo and in-vitro study on the guinea-pig ileum. *Archives Internationales de Pharmacodynamie et de Thérapie*.

[47] Riyazi A., Hensel A., Bauer K., Geißler N., Schaaf S., Verspohl E. (2007). The effect of the volatile oil from ginger rhizomes (Zingiber officinale), its fractions and isolated compounds on the 5-HT3 receptor complex and the serotoninergic system of the rat ileum. *Planta Medica*.

[48] Abu-Niaaj L., Abu-Zarga M., Sabri S., Abdalla S. (1993). Isolation and biological effects of 7-O-methyleriodictyol, a flavanone isolated fromArtemisia monosperma, on rat isolated smooth muscles. *Planta Medica*.

[49] Krenn L., Beyer G., Pertz H. H. (2004). In vitro antispasmodic and antiinflammatory effects of Drosera rotundifolia. *Arzneimittelforschung*.

[50] Alexandrovich I., Rakovitskaya O., Kolmo E., Sidorova T., Shushunov S. (2003). The effect of fennel (Foeniculum vulgare) seed oil emulsion in infantile colic: a randomized, placebo-controlled study. *Alternative Therapies in Health and Medicine*.

[51] Mengistu M., Abebe Y., Mekonnen Y., Tolessa T. (2012). In vivo and in vitro hypotensive effect of aqueous extract of Moringa stenopetala. *African Health Sciences*.

[52] Yazdi H., Seifi A., Changizi S. (2017). Hydro-alcoholic extract of Matricaria recutita exhibited dual anti-spasmodic effect via modulation of Ca^2+^ channels, NO and PKA2-kinase pathway in rabbit jejunum. *Avicenna Journal of Phytomedicine*.

[53] Achterrath-Tuckermann U., Kunde R., Flaskamp E., Isaac O., Thiemer K. (1980). Pharmakologische Untersuchungen von Kamillen-Inhaltsstoffen. *Planta Medica*.

[54] Mehmood M. H., Munir S., Khalid U. A., Asrar M., Gilani A. H. (2015). Antidiarrhoeal, antisecretory and antispasmodic activities of Matricaria chamomilla are mediated predominantly through K(+)-channels activation. *BMC Complementary Medicine and Therapies*.

[55] Mehmood M. H., Gilani A. H. (2010). Pharmacological basis for the medicinal use of black pepper and piperine in gastrointestinal disorders. *Journal of Medicinal Food*.

[56] Gilani A. H., Mehmood M. H., Janbaz K. H., Khan A. U., Saeed S. A. (2008). Ethnopharmacological studies on antispasmodic and antiplatelet activities of *Ficus carica*. *Journal of Ethnopharmacology*.

[57] Godfraind T., Miller R., Wibo M. (1986). Calcium antagonism and Ca++ entry blockade. *Physiological Reviews*.

[58] Choi E. M., Hwang J. K. (2004). Antiinflammatory, analgesic and antioxidant activities of the fruit of *Foeniculum vulgare*. *Fitoterapia*.

[59] Duško B. L., Comiæ L., Sukdolak S. (2006). Antibacterial activity of some plants from family Apiaceae in relation to selected phytopathogenic bacteria. *Kragujevac Journal of Science*.

[60] Parejo I., Jauregui O., Sánchez-Rabaneda F., Viladomat F., Bastida J., Codina C. (2004). Separation and characterization of phenolic compounds in fennel (Foeniculum vulgare) using liquid chromatography-negative electrospray ionization tandem mass spectrometry. *Journal of Agricultural and Food Chemistry*.

[61] Scalbert A., Manach C., Morand C., Rémésy C., Jiménez L. (2005). Dietary polyphenols and the prevention of diseases. *Critical Reviews in Food Science and Nutrition*.

[62] Rahimi R., Ardekani M. R. S. (2013). Medicinal properties of Foeniculum vulgare Mill. in traditional Iranian medicine and modern phytotherapy. *Chinese Journal of Integrative Medicine*.

[63] Badgujar S. B., Patel V. V., Bandivdekar A. H. (2014). Foeniculum vulgare Mill: a review of its botany, phytochemistry, pharmacology, contemporary application, and toxicology. *BioMed Research International*.

[64] Boskabady M., Khatami A., Nazari A. (2004). Possible mechanism (s) for relaxant effects of Foeniculum vulgare on guinea pig tracheal chains. *Die Pharmazie-An International Journal of Pharmaceutical Sciences*.

[65] Ostad S., Soodi M., Shariffzadeh M., Khorshidi N., Marzban H. (2001). The effect of fennel essential oil on uterine contraction as a model for dysmenorrhea, pharmacology and toxicology study. *Journal of Ethnopharmacology*.

[66] Nickavar B., Mojab F., Mojahedi A. (2009). Composition of the essential oil from Anthriscus nemorosa. *Chemistry of Natural Compounds*.

[67] Sadraei H., Asghari G., Hekmatti A. A. (2003). Antispasmodic effect of three fractions of hydroalcoholic extract of *Pycnocycla spinosa*. *Journal of Ethnopharmacology*.

[68] Harborne A. (1998). *Phytochemical Methods a Guide to Modern Techniques of Plant Analysis*.

[69] Hajhashemi V., Sadraei H., Ghannadi A. R., Mohseni M. (2000). Antispasmodic and anti-diarrhoeal effect of *Satureja hortensis* L. essential oil. *Journal of Ethnopharmacology*.

[70] Clissold S. P., Heel R. C. (1985). Transdermal hyoscine (scopolamine) A Preliminary Review of its Pharmacodynamic Properties and Therapeutic Efficacy. *Drugs*.

[71] Izaddoost M., Robinson T. (1987). *Synergism and antagonism in the pharmacology of alkaloidal plants. Herbs, spices, and medicinal plants: recent advances in botany, horticulture, and pharmacology (USA)*.

[72] Kang P., Seol G. H. (2015). Linalool elicits vasorelaxation of mouse aortae through activation of guanylyl cyclase and K+ channels. *Journal of Pharmacy and Pharmacology*.

[73] Lis-Balchin M., Hart S. Pharmacological effect of essential oils on the uterus compared to that on other tissue types. *Proceedings of Proceedings of the 27th International Symposium on Essential Oils, Vienna, Austria*.

[74] Reiter M., Brandt W. (1985). Relaxant effects on tracheal and ileal smooth muscles of the guinea pig. *Arzneimittel-Forschung*.

[75] Aguilar Contreras A. (1994). *Herbario Medicinal del Instituto Mexicano del Seguro Social: Información Etnobotánica*.

[76] Jia J., Li Y., Lei Z. (2013). Relaxative effect of core licorice aqueous extract on mouse isolated uterine horns. *Pharmaceutical Biology*.

[77] Lee K. K., Omiya Y., Yuzurihara M., Kase Y., Kobayashi H. (2013). Antispasmodic effect of shakuyakukanzoto extract on experimental muscle cramps *in vivo*: Role of the active constituents of Glycyrrhizae radix. *Journal of Ethnopharmacology*.

[78] Nagai H., He J. X., Tani T., Akao T. (2007). Antispasmodic activity of licochalcone A, a species-specific ingredient of Glycyrrhiza inflata roots. *Journal of Pharmacy and Pharmacology*.

[79] Yazdanparast R., Bahramikia S. (2008). Evaluation of the effect of Anethum graveolens L. crude extracts on serum lipids and lipoproteins profiles in hypercholesterolaemic rats. *DARU Journal of Pharmaceutical Sciences*.

[80] Naseri M. G., Heidari A. (2007). Antispasmodic effect of Anethum graveolens fruit extract on rat ileum. *International Journal of Pharmacology*.

[81] Sagar K., U S., GR A. R., Mohan K., Narendran G. (2018). Effect of swarnamritaprashana on growth and development in Indian toddlers. *International Journal of Research in Ayurveda and Pharmacy*.

[82] Heidarifar R., Mehran N., Heidari A., Koohbor M., Mansourabad M. K. (2014). Effect of Dill (Anethum graveolens) on the severity of primary dysmenorrhea in compared with mefenamic acid: a randomized, double-blind trial. *Journal of Research in Medical Sciences: the Official Journal of Isfahan University of Medical Sciences*.

[83] Gebhardt Y., Witte S., Forkmann G., Lukačin R., Matern U., Martens S. (2005). Molecular evolution of flavonoid dioxygenases in the family Apiaceae. *Phytochemistry*.

[84] Möhle B., Heller W., Wellmann E. (1985). UV-Induced biosynthesis of quercetin 3-*O*-*β*-D-glucuronide in dill cell cultures. *Phytochemistry*.

[85] Cimanga R., Mukenyi P. N. K., Kambu O. K. (2010). The spasmolytic activity of extracts and some isolated compounds from the leaves of *Morinda morindoides* (Baker) Milne-Redh. (Rubiaceae). *Journal of Ethnopharmacology*.

[86] Qari S. H. (2008). *In vitro* evaluation of the anti-mutagenic effect of *Origanum majorana* extract on the meristemetic root cells of *Vicia faba*. *Journal of Taibah University for Science*.

[87] Suhaj M. (2006). Spice antioxidants isolation and their antiradical activity: a review. *Journal of Food Composition and Analysis*.

[88] Abdel-Massih R. M., Fares R., Bazzi S., el-Chami N., Baydoun E. (2010). The apoptotic and anti-proliferative activity of *Origanum majorana* extracts on human leukemic cell line. *Leukemia Research*.

[89] Vági E., Rapavi E., Hadolin M. (2005). Phenolic and triterpenoid antioxidants from Origanum majorana L. herb and extracts obtained with different solvents. *Journal of Agricultural and Food Chemistry*.

[90] Pimple B., Kadam P., Patil M. (2012). Comparative antihyperglycaemic and antihyperlipidemic effect of Origanum majorana extracts in NIDDM rats. *Oriental Pharmacy and Experimental Medicine*.

[91] Pimple B., Kadam P. V., Patil M. J. (2012). Ulcer healing properties of different extracts of *Origanum majorana* in streptozotocin-nicotinamide induced diabetic rats. *Asian Pacific journal of Tropical Disease*.

[92] Mohamoud I. A. A., Idris F. O., Adam S. I. Y. (2020). Antidiabetic Activity of Origanum Majorana L in Glucose Fed Normal Rats and Alloxan-Induced Diabetic Rats. *Sudan Journal of Science and Technology*.

[93] Mawa S., Husain K., Jantan I. (2013). Ficus carica L. (Moraceae): Phytochemistry, Traditional Uses and Biological Activities. *Evidence-based Complementary and Alternative Medicine*.

[94] Ma Y.-M., Liang X. A., Zhang H. C., Liu R. (2016). Cytotoxic and antibiotic cyclic pentapeptide from an endophytic Aspergillus tamarii of Ficus carica. *Journal of Agricultural and Food Chemistry*.

[95] Mahboubi M., Kazempour N. (2011). Chemical composition and antimicrobial activity of Satureja hortensis and Trachyspermum copticum essential oil. *Iranian Journal of Microbiology*.

[96] Harmati M., Gyukity-Sebestyen E., Dobra G. (2017). Binary mixture of *Satureja hortensis* and *Origanum vulgaresub* sp.hirtumessential oils: in vivo therapeutic efficiency against *Helicobacter pylori* infection. *Helicobacter*.

[97] Qiao D., Gan L. S., Mo J. X., Zhou C. X. (2012). Chemical constituents of Acorus calamus. *Zhongguo Zhong yao za zhi = Zhongguo zhongyao zazhi = China journal of Chinese materia medica*.

[98] Rajput S. B., Tonge M. B., Karuppayil S. M. (2014). An overview on traditional uses and pharmacological profile of *Acorus calamus* Linn. (Sweet flag) and other *Acorus* species. *Phytomedicine : International Journal of Phytotherapy and Phytopharmacology*.

[99] Gilani A. U. H., Shah A. J., Ahmad M., Shaheen F. (2006). Antispasmodic effect of Acorus calamus Linn. is mediated through calcium channel blockade. *Phytotherapy Research: An International Journal Devoted to Pharmacological and Toxicological Evaluation of Natural Product Derivatives*.

[100] Tanideh N., Jamshidzadeh A., Sepehrimanesh M. (2016). Healing acceleration of acetic acid-induced colitis by marigold (Calendula officinalis) in male rats. *Saudi Journal of Gastroenterology: Official Journal of the Saudi Gastroenterology Association*.

[101] Bashir S., Janbaz K. H., Jabeen Q., Gilani A. H. (2006). Studies on spasmogenic and spasmolytic activities of Calendula officinalis flowers. *Phytotherapy Research: An International Journal Devoted to Pharmacological and Toxicological Evaluation of Natural Product Derivatives*.

[102] Laribi B., Kouki K., Mougou A., Marzouk B. (2010). Fatty acid and essential oil composition of three Tunisian caraway (Carum carvi L.) seed ecotypes. *Journal of the Science of Food and Agriculture*.

[103] Showraki A., Emamghoreishi M., Oftadegan S. (2016). Anticonvulsant effect of the aqueous extract and essential oil of Carum carvi L. seeds in a pentylenetetrazol model of seizure in mice. *Iranian Journal of Medical Sciences*.

[104] Kazemipoor M., Hamzah S., Hajifaraji M., Radzi C. W. J. B. W. M., Cordell G. A. (2016). Slimming and appetite-suppressing effects ofCarawayAqueous extract as a natural therapy in physically active women. *Phytotherapy Research*.

[105] al-Essa M. K., Shafagoj Y. A., Mohammed F. I., Afifi F. U. (2010). Relaxant effect of ethanol extract of Carum carvi on dispersed intestinal smooth muscle cells of the guinea pig. *Pharmaceutical Biology*.

[106] Fu H., Luo Y., Zhang D. (2009). Studies on chemical constituents of leaves of Psidium guajava. *Zhongguo Zhong yao za zhi= Zhongguo zhongyao zazhi=China Journal of Chinese Materia Medica*.

[107] Morais-Braga M. F. B., Sales D. L., Carneiro J. N. P. (2016). *Psidium guajava* L. and *Psidium brownianum* Mart ex DC.: Chemical composition and anti - *Candida* effect in association with fluconazole. *Microbial Pathogenesis*.

[108] Lozoya X., Reyes-Morales H., Chávez-Soto M. A., Martínez-García M.´. . C., Soto-González Y., Doubova S. V. (2002). Intestinal anti-spasmodic effect of a phytodrug of *Psidium guajava folia* in the treatment of acute diarrheic disease. *Journal of Ethnopharmacology*.

[109] Amel B., Cherif H. S., Eswayah A. A., Abdennour M. A., Oliveira I. V. (2021). Evaluation of the anti-inflammatory, antispasmodic and healing effects of walnut leaves Juglans regia L. aqueous extract. *Arabian Journal of Medicinal and Aromatic Plants*.

[110] Vanegas Andrade C., Matera S., Bayley M. (2021). Antispasmodic, antidepressant and anxiolytic effects of extracts from *Schinus lentiscifolius* Marchand leaves. *Journal of Traditional and Complementary Medicine*.

[111] Roome T., Qasim M., Farooq A. D., Ilyas Q., Aziz S., Ali S. F. (2021). Antispasmodic activity and mechanism of action of polyherbal formulation DCD-684 on rabbit jejunum. *Pakistan Journal of Pharmaceutical Sciences*.

[112] Parcha P. K., Sarvagalla S., Ashok C. (2021). Repositioning antispasmodic drug Papaverine for the treatment of chronic myeloid leukemia. *Pharmacological Reports*.

[113] Shamkuwar P. (2021). Evaluation of antispasmodic potential of polyherbal formulation. *Asian Journal of Biological and Life Sciences*.

[114] Chinnappan S., Mogana R., Qin T. X. (2020). In vitroantimotility and antispasmodic effects ofNephelium lappaceumon isolated chicken ileum. *Research Journal of Pharmacy and Technology*.

[115] Sadraei H., Sajjadi S. E., Tarafdar A. (2020). Antispasmodic effect of hydroalcoholic and flavonoids extracts of *Dracocephalum kotschyi* on rabbit bladder. *J Herbmed Pharmacol*.

[116] Lima C. C., de Holanda-Angelin-Alves C. M., Pereira-Gonçalves Á. (2020). Antispasmodic effects of the essential oil of *Croton zehnteneri*, anethole, and estragole, on tracheal smooth muscle. *Heliyon*.

[117] Mbugua P. M., Oluoch L. L., Chege B. M., Siringo C. G., Wangechi A. M. (2020). *The antispasmodic effect of aqueous root bark extract of Carissa edulis (Forssk.) Vahl on isolated rabbit jejunum is mediated through blockade of calcium channels*.

[118] Milutinović M., Branković S., Ćujić N. (2020). Antispasmodic effects of black chokeberry (Aronia melanocarpa (Michx.) Elliott) extracts and juice and their potential use in gastrointestinal disorders. *Journal of Berry Research*.

[119] Memon A. H., Tan M. H., Khan M. S. S. (2020). Toxicological, antidiarrhoeal and antispasmodic activities of Syzygium myrtifolium. *Revista Brasileira de Farmacognosia*.

[120] Ansari M. N. (2020). Assessment of antidiarrheal, antispasmodic and antimicrobial activities of methanolic seeds extract of Peganum harmala L.(Nitrariaceae). *Journal of Pharmaceutical Research International*.

[121] Monteiro F. . S., Costa J. R. . S., Martins L. J. A., Rocha C. Q. ., Borges A. C. R., Borges M. O. . R. (2020). Hydroalcoholic extract of leaves of Arrabidaea brachypoda (DC.) Bureau present antispasmodic activity mediated through calcium influx blockage. *Revista de Ciências Farmacêuticas Básica e Aplicada*.

[122] Heghes S. C., Vostinaru O., Rus L. M., Mogosan C., Iuga C. A., Filip L. (2019). Antispasmodic effect of essential oils and their constituents: a review. *Molecules*.

[123] Zoofishan Z., Kúsz N., Csorba A. (2019). Antispasmodic activity of prenylated phenolic compounds from the root bark of Morus nigra. *Molecules*.

[124] Beyi L., Zrouri H., Makrane H. (2021). Relaxant and antispasmodic activities of aqueous extract from Thymus algeriensis Boiss. and Reut. *Journal of Natural Remedies*.

[125] Kassim D., Ouattara G. A., David A. D. S., Benoit N.’g. B., Paul Y. A. (2020). The antispasmodic activity of ethanol extract of the stem bark of Piliostigma reticulatum Horscht D.C (Ceasalpiniaceae), and its dichloromethane fraction isolated. *GSC Biological and Pharmaceutical Sciences*.

